# Editorial: Essential metals for plants: Uptake, transport, regulation of homeostasis and roles in plant development

**DOI:** 10.3389/fpls.2023.1156247

**Published:** 2023-02-15

**Authors:** Sidsel Birkelund Schmidt, Olena Vatamaniuk, Anja Schneider

**Affiliations:** ^1^ Innovation Centre for Organic Farming, Aarhus, Denmark; ^2^ Soil and Crop Sciences Section, Cornell University, Ithaca, NY, United States; ^3^ Plant Science Group, Faculty of Biology, Ludwig-Maximilian University of Munich, Munich, Germany

**Keywords:** copper, iron, zinc, stable isotope fractionation, metal deficiency, rice breeding, nutrient uptake, Arabidopsis

Metals are needed by all living organisms to function properly and in plants they are taken up from the soil. In both natural and anthropogenic soils, plants are exposed to a wide range of metal concentrations. Plants have therefore evolved sophisticated mechanisms to cope with fluctuating levels of metal supply from deficiency to toxicity. Metalloproteins comprise almost half of all naturally occurring proteins, which underlines the importance of a balanced and sufficient metal availability. Among the most complex reactions in a plant cell, photosynthesis, respiration, and nitrogen fixation strictly depend on metals as catalysts and cofactors. Plants can regulate the balance of metals in the cell, to ensure sufficient supply of metals to maintain substantial reactions and on the other hand, to protect the cell from damage and toxicity by allocating harmful metals. The nine articles collected for this Research Topic describe different aspects of metal homeostasis, with a special focus on the essential metals copper (Cu), iron (Fe) and zinc (Zn).

In the review by Wiggenhauser et al. recommendations are given to better exploit the information provided by varying isotope compositions, to further advance the non-traditional isotope process tracing of metals in plants. Isotope fractionation describes the small changes in relative isotope abundances during chemical reactions or physical processes. Several processes drive isotope fractionation in plants and for instance, changes in nutrient supply induced shifts in isotope fractionation patterns for Mg, Cu and Zn, suggesting that isotope process tracing can be used as a tool to provide information on acquisition mechanisms, e.g. active or passive uptake. Using this technique, Blotevogel et al. analyzed stable Cu isotope ratios in Grapevine (*Vitis vinifera*). It turned out, that there was no direct relationship between Cu contents in soils or soil solutions and Cu contents in roots, indicating a partly homeostatic control of Cu uptake. However, at low Cu supply, the Cu-isotope fractionation between soil solution and roots showed that isotopically light Cu was preferentially taken up whereas heavy Cu isotopes were increasingly taken up at high Cu supply levels, suggesting a shift from active to passive uptake mechanisms. The authors emphasize that Cu isotope analysis is a sensitive tool to monitor differences in Cu uptake and translocation pathways even before differences in tissue contents become obvious.

The work by Mancini and Garcia-Molina describes the consequences of simultaneous Cu and Fe deficiency in *Arabidopsis thaliana* with a particular investigation of alternative splicing events on the transcriptome of rosette leaves. Functional annotation of transcript changes detected under Fe and Cu deficiency revealed that differently expressed genes belong to general stress responses and the translation machinery, while differently spliced genes belong to metabolic reactions, more specifically amino acid biosynthesis. The authors propose that during systematic reprogramming, alternative splicing could act as an independent mechanism to regulate metabolic reactions, which are not adjusted at the transcript or protein level. In a minireview by Su et al. the aspect of chromatin-based regulation in response to the plant Fe status is highlighted and involves histone modification and DNA methylation. Histone trimethylation cannot only induces chromatin condensation resulting in gene silencing during Fe sufficient conditions, but can also activate corresponding target genes in Fe deficient conditions in *Arabidopsis thaliana.* In rice plants, DNA hypermethylation, in the promotor region of corresponding downstream genes, leads to enhanced transcription during Fe deficiency, whereas the unmethylated stage confers a basal expression of Fe homeostasis genes.

A useful breeding line is described by Kandwal et al. which contain high concentrations of Fe and Zn in the rice grain, without affecting growth in a paddy field. This rice line possessed a non-sense mutation of the vacuolar localized Fe transporter OsVIT2 and thus Fe and Zn relocation changed in benefit of the grain. The line used in that study is an EMS-mutagenized rice line and therefore offers many advantages compared to transgenic approaches. It highlights the use of OsVIT2 mutations in marker-assisted breeding programs for future grain biofortification of Fe and Zn. To develop strategies for improving Zn content in legume crops, the mechanisms of response to Zn deficiency in the legume model *Medicago truncatula* was investigated by Liao et al. In legumes species, an adequate supply of Zn to the nodule-rhizobia infected cells is required to ensure optimal symbiotic nitrogen fixation. Two F-group basic region leucine-zipper (F-bZip) transcription factors were described and functionally characterized in *M. truncatula*, and MtFbZIP1 was identified as a key regulator of the Zn deficiency response related gene expression. Another approach to overcome crop Zn deficiency is described by Li et al. where the authors studied foliar Zn fertilization in sunflower leaves and subsequent translocation by transcriptome analysis combined with synchrotron-based X-ray fluorescence microscopy (XFM). It was observed that foliar Zn application caused rapid stress to the leaf, but once the stress decreased Zn was moved from the leaf epidermis to the vascular tissues. However, it was proposed that loading of Zn into the phloem, or the limited Zn mobility within the phloem itself, determine the restricted translocation of foliar applied Zn in sunflower leaves.

In terrestrial plants nutrients are mainly taken up through the roots. The Casparian strip represents a physical barrier controlling the inward flow of water and nutrients. Yang et al. reports of a novel rice mutant with delayed Casparian strip formation and ectopic suberin depositions in small lateral roots. As a consequence, the mutant showed higher concentrations of Fe, manganese (Mn) and sodium (Na) and a reduced tolerance to salt stress due to altered ion permeability. The affected gene encodes a Casparian strip membrane domain protein, which may form a transmembrane scaffold to recruit lignin biosynthetic enzymes.

In the water ecosystem nutrient uptake *via* the whole plant of submerged macrophytes is described by Xian et al. by comparing two species with different tolerances to ammonium. Ammonium uptake through the above-ground part was dominant under excess ammonium conditions, and the tolerant species possessed a higher plasticity in ammonium utilization under various concentrations and is a good candidate for applications in phytoremediation of polluted water.

## Author contributions

All authors listed have made a substantial, direct and intellectual contribution to the work, and approved it for publication.

